# Outcome assessment of emergency laparotomies and associated factors in low resource setting. A case series

**DOI:** 10.1016/j.amsu.2018.09.029

**Published:** 2018-09-25

**Authors:** Endale Gebreegziabher Gebremedhn, Abatneh Feleke Agegnehu, Bernard Bradley Anderson

**Affiliations:** a Department of Anaesthesia, School of Medicine, Gondar College of Medicine and Health Sciences, The University of Gondar, Gondar, Ethiopia; b Department of Surgery, School of Medicine, Gondar College of Medicine and Health Sciences, The University of Gondar, Gondar, Ethiopia

**Keywords:** Emergency laparotomy, Morbidity, Mortality, Associated factors, Low resource setting

## Abstract

**Background:**

Emergency laparotomy is a high risk procedure which is demonstrated by high morbidity and mortality. However, the problem is tremendous in resource limited settings and there is limited data on patient outcome. We aimed to assess postoperative patient outcome after emergency laparotomy and associated factors.

**Methods:**

An observational study was conducted in our hospital from March 11- June 30, 2015 using emergency laparotomy network tool. All consecutive surgical patients who underwent emergency laparotomy were included. Binary and multiple logistic regressions were employed using adjusted odds ratios and 95% CI, and P-value < 0.05 was considered to be statistically significant.

**Result:**

A total of 260 patients were included in the study. The majority of patients had late presentation (>6hrs) to the hospital after the onset of symptoms of the diseases and surgical intervention after hospital admission. The incidences of postoperative morbidity and mortality were 39.2% and 3.5% respectively. Factors associated with postoperative morbidity were preoperative co-morbidity (AOR = 0.383, CI = 0.156–0.939) and bowel resection (AOR = 0.232, CI = 0.091–0.591). Factors associated with postoperative mortality were anesthetists' preoperative opinion on postoperative patient outcome (AOR = 0.067, CI = 0.008–0.564), level of consciousness during recovery from anaesthesia (AOR = 0.114, CI = 0.021–10.628) and any re-intervention within 30 days after primary operation (AOR = 0.083, CI = 0.009–0.750).

**Conclusion and recommendation:**

The incidence of postoperative morbidity and mortality after emergency laparotomy were high. We recommend preoperative optimization, early surgical intervention, and involvement of senior professionals during operation in these risky surgical patients. Also, we recommend the use of WHO or equivalent Surgical Safety Checklist and establishment of perioperative patient care bundle including surgical ICU and radiology investigation modalities such as CT scan.

## Highlights

• Emergency laparotomy caused morbidity and mortality in 39.2% and 3.5% patients respectively.
• Large bowel resection and re-operation contributed most to morbidity and mortality.
• Delayed arrival at hospital and delayed surgical interventions were the main problems.

## 1 Introduction

Emergency laparotomy (EP) is a common procedure which associated with substantial postoperative morbidity and mortality [1,2]. Compared with other acute surgical emergencies, patients undergoing emergency laparotomy have a disproportionately high mortality both in younger [3,4] and older sick patients [5]. EP is a resource-intensive surgical procedure with a high morbidity and mortality rates even in the best healthcare systems and remain an area of focus for quality improvement in developed nations [[6], [7], [8]]. Perioperative management of patients undergoing emergency laparotomy in middle and low-income countries is extremely challenging, and causes high postoperative 30-day patient morbidity and mortality as well as imposes a high healthcare cost burden [9]. Despite this, there is paucity of evidence on postoperative patient morbidity and mortality after emergency laparotomy in resource-limited settings which hamper the establishment of evidence-based optimal perioperative care bundle [9]. In addition, in low-income countries, there are large volumes of emergency patients who need surgical care. However, infrastructures such as operation rooms, advanced equipment, skilled human resources, investigation modalities such as Computerized tomography (CT) scan, Magnetic Resonance Imaging (MRI), Ultrasound (US) and drugs are limited [9]. Moreover, even with the available resources, there are variations in the preoperative patient optimization, surgical/anaesthetic quality care provision and utilization of the available resources all of which could negatively impact on postoperative patient outcome [9].

In this study, we characterized the heterogeneity of patients presented with acute abdomen, underlined pathologies, delay from the onset of symptoms of the diseases to hospital admission and surgical interventions, types of surgical interventions performed, and postoperative morbidity and mortality within 30 days of emergency laparotomy in a tertiary teaching and referral governmental hospital with a high load of emergency patients with limited resources for patient care.

## 2 Methods

### 2.1 Registration and ethics

Ethical approval was obtained from College of Medicine and Health Sciences, Academic, Research and Community Services Vice Dean (Ref.No.CMHS248/07). This study was also registered in researchregistry.com (researchregistry3317). Oral informed consent was obtained from each study subject after explanation of what they will take part in the research and any involvement was after their complete consent. Anyone who was not willing to participate in the study had full right not to participate. Confidentiality was ensured from all the data collectors and investigators using anonymous questionnaire and keeping questionnaires locked. This work has been reported in line with the PROCSS criteria [10].

### 2.2 Study design

This is a single centre prospective observational study. All consecutive patients (cases) who underwent emergency laparotomy during the study period were included.

### 2.3 Setting

This is one of the largest governmental tertiary Teaching and Referral hospitals in the country which provides health services for more than five million people in the catchment area. The hospital has 500 hundred beds, seven operation theatres, one medical and one paediatrics intensive care units. The study conducted from March 11-June 30, 2015. Data was collected using Emergency Laparotomy Network tool. A pre-tested, structured, English version questionnaire and checklist used to collect the data (developed based on Emergency Laparotomy Network Tool; https://data.nela.org.uk). The English version questionnaire was pre-tested before actual data collection. One BSc holder data collector was selected and one day training was given to complete data collection. Training of data the collector and pre testing activities were took place from February 15–30, 2015.

To ensure the quality of data, training was given for data collectors and the investigators have been directing and monitor the whole data collection processes for consistency, completeness and accuracy. Pre-test was done; data cleaned and checked every day, and double data entry technique used during data entry.

### 2.4 Participants

All consecutive patients who underwent emergency laparotomies in our hospital during the study period were included. Whereas cholecystitis or internal hernia after gastric bypass which in the local setting are treated as a semi-acute setup, laparotomies for non-planned reoperations after recent surgical procedures and primary acute laparotomies in patients operated more than 24 h post admission (in order to exclude patients with conditions that did not warrant immediate surgery) were excluded from the study.

#### 2.4.1 Study variables

The main outcomes of interest were postoperative complication, and death. The sociodemographic variables were age, sex, body mass index (BMI), American Anesthesiologists’ (ASA) status, preoperative complication, preoperative co-morbidity, surgical indication, seniority of anaesthetist and surgeon, length of hospital stay, perioperative temperature, time of patient admission. In addition, anaesthesia related factors also include: type of anaesthesia: General anaesthesia (Laryngeal mask airway, endotracheal intubation, sedation: intravenous anaesthesia, inhalational anaesthesia) vs regional anaesthesia (spinal, epidural, Caudal, peripheral neve block), anaesthetic related complication, premedication. Moreover, operation related factor comprised of indication for surgery, type of operation (general surgery: colorectal, pancreatic, gastric surgery & Urological: cystectomy, prostatectomy and nephrectomy), extent of operation (minor, major), risk of operation (low or high), duration of surgery, specific type of operation, timing of surgery (early vs late). Furthermore, place of postoperative patient follow up, postoperative complications and postoperative death (time, cause for death) were assessed.

### 2.5 Operational definitions

**Emergency:** Immediate lifesaving operation, resuscitation simultaneous with surgical treatment (operation usually within 1hr).

**Emergency laparotomy:** Emergency operation which involves exploration of the abdomen.

**Postoperative mortality:** Defined as death within 30 days after primary emergency laparotomy.

**Postoperative morbidity:** Defined as operation related complications that occurred within 30 days after operation.

**Major operation:** Defined as any invasive operative procedure in which a more extensive resection is performed, e.g. a body cavity is entered, organs are removed, or normal anatomy is altered-in general, if a mesenchymal barrier was opened (pleural cavity, peritoneum, meninges).

**Minor operation:** A minor operation was defined as any invasive operative procedure in which only skin or mucus membranes and connective tissue are resected, e.g. vascular cut-down for catheter placement or implanting pumps in subcutaneous tissue.

### 2.6 Statistical analysis

The data coded, entered and analyzed using SPSS version 20 software. Associations between dependent and independent variables were assessed and its strength was presented using adjusted odds ratios and 95% confidence interval. Binary and multiple logistic regressions were used to assess the association between outcome and explanatory variables. Variables from the bivariate analysis were fitted for the two outcome variables in relation to each explanatory variable. Those variables which will fulfil the minimum requirement of 0.2 level of significance were further entered in to multivariate logistic regression analysis for further assessment and the fitness of model the was checked using Hosmer and Lemeshow goodness of fitness. Frequency tables, graphs and summary statistics were used.

## 3 Result

### 3.1 Socio-demographic characteristics of the study participants

A total of 260 patients were included in the study with a response rate of 100%. Of the study participants, 167 (64.2%) were males. The majority of patients were American Society of Anesthesiologists' Physical Status three (ASA3: n = 188, 72.3%) whereas ASA2 (n = 36, 13.8%), ASA4 (n = 23, 8.8%), and ASA1 (n = 12, 4.6%) respectively. Thirty three out of 260 (12.7%) patients had preoperative associated co-morbidities [Table 1].

**Table 1:** Socio-demographic characteristics of the study participants (N = 260), 2015.

Factor	Frequency	Percentage (%)
Age (year)		
<1	5	1.9
1–18	65	25
19–29	79	30.4
30–45	65	25
50–65	34	13.1
>65	12	4.6
Co-morbidity		
No	227	87.3
Yes	35	12.7
Smoking history		
Current	6	2.3
Previous	9	3.5
Never	245	94.2
Preoperative CT		
No	260	100
Yes	0	0
Preoperative Hgb		
Yes	212	81.5
No	48	18.5
V/S at admission		
Stable	234	90
Unstable	26	10
Premedication		
No	141	54.2
Yes	119	45.8
Preop analgesia		
No	217	83.5
Yes	43	16.5

None of the patients had CT scanning before surgery as CT was not available in the hospital during the study period.

### 3.2 Type of anaesthesia and factors related with anaesthesia

The majority of patients (n = 248, 95.4%) were operated upon under general anaesthesia with endotracheal intubation whereas 12 (4.6%) were operated upon under combined general and regional anaesthesia respectively. Two hundred and twenty four (86.2%) patients were induced with ketamine whereas 16 (6.2%), 7 (2.7%), and 1 (0.4%) of patients were induced with thiopentone, propofol and halothane respectively. Suxamethonium was used for intubation for the majority of patients (n = 241, 92.7%) followed by pancuronium 5 (1.9%) and vecuronium 2 (0.8%) respectively.

Two hundred and forty (92.3%) patients were maintained with halothane during operation whereas 7 (2.7%), 1 (0.4%) and 12 (4.6%) patients were maintained with intravenous drugs, combined intravenous and inhalational anaesthetics, and preoperatively instituted regional anaesthesia such as epidural anaesthesia respectively. Most patients were monitored with pulseoximetry, non-invasive blood pressure apparatus and ECG during operation. There was no capnograph during the study period [Table 2].

**Table 2:** Factors related with anaesthesia (N = 260), 2015.

Factor	Frequency	Percentage (%)
Qualification of anaesthetist		
BSc	164	63.1
MSc student	53	20.4
MSc	43	16.5
Monitoring used		
Pulseoximetry alone	2	0.8
NIBP, pulseoximetry	9	3.5
NIBP,ECG, pulseoximetry	249	95.8
Intraoperative analgesia		
Systemic	224	86.2
Regional	12	4.6
No	25	9.6
Intraoperative warming		
Blanket	259	0.4
Heater	1	99.6
V/S during recovery phase		
Stable	258	99.8
Unstable	2	0.2
Consciousness level during recovery from anaesthesia		
Fully awake	213	81.9
Half awake	43	16.5
Not awake	4	1.5

The majority of patients (n = 111, 42.7%) were given 2 L of fluid during operation whereas 29 (11.2%), 76 (29.2%), 29 (24 (9.2%), 5 (1.9%), and 2 (0.8%) patients were given < 1 L, 1 L, 3 L, 4 L and 5 L respectively with the mean value of 1.6 L. Three patients were not given fluid intraoperatively.

### 3.3 Type of surgery and factors related with surgery

One hundred and sixty one (n = 161, 61.9%) out of 260 patients had undergone abdominal operation followed by appendectomy [Table 3].

**Table 3:** Factors related with surgery (N = 260), 2015.

Factor	Frequency	Percentage (%)
Surgeon		
Senior involved in operation	84	32.3
Senior resident with consultation	176	67.7
Type of operation		
Laparotomy	161	61.9
Appendectomy	99	38.1
Surgical incision type		
Midline	129	49.6
Transverse	10	3.8
Lanz	94	36.2
Groin	14	5.4
Rooftop	5	1.9
Kocker's	8	3.1
Bowel resection		
No	221	85
Yes – handsewn anastomosis	31	11.9
Yes – stoma without anastomosis	6	2.3
Yes – stapled anastomosis	0	0
Other	2	0.8
Stoma formation		
None	241	92.7
Loop ileostomy	0	0
Loop colostomy	6	2.3
End ileostomy	2	0.8
End colostomy	11	4.2

**Fig. 1:**
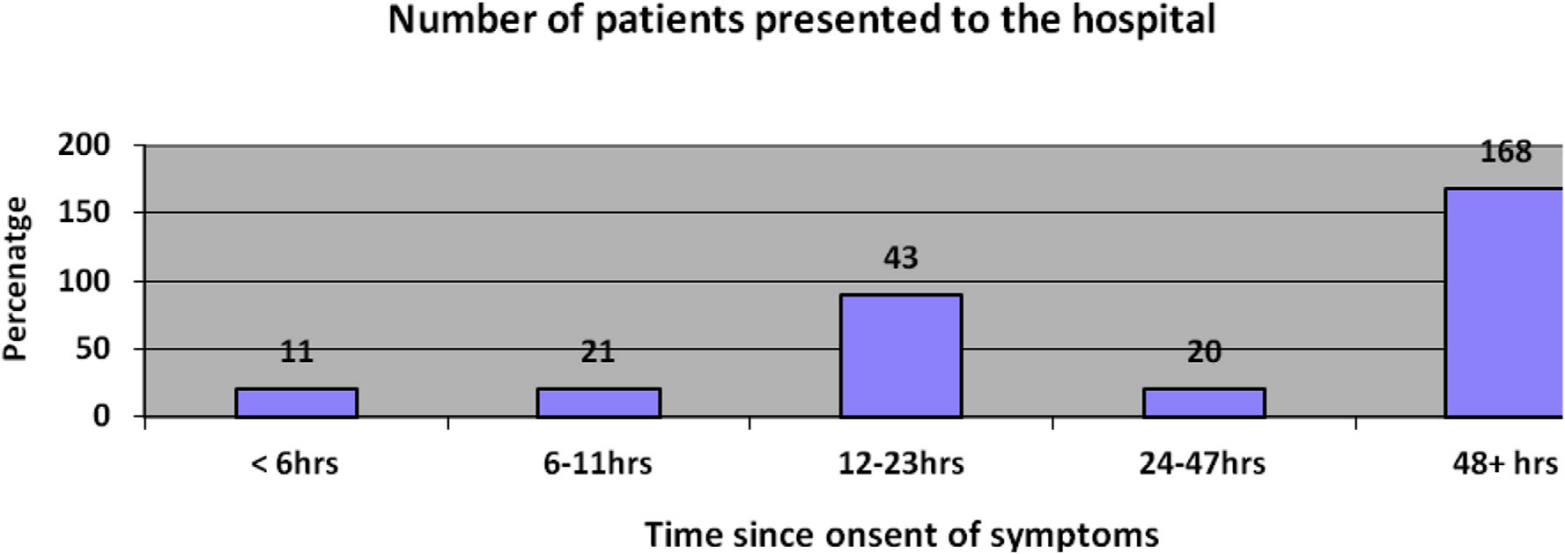
Presentation of patients to the hospital after the onset of the symptoms of the diseases, 2015.

The majority of patients had late (>6hrs) presentation to the hospital after the onset of symptoms of the diseases [Fig. 1.].

**Fig. 2:**
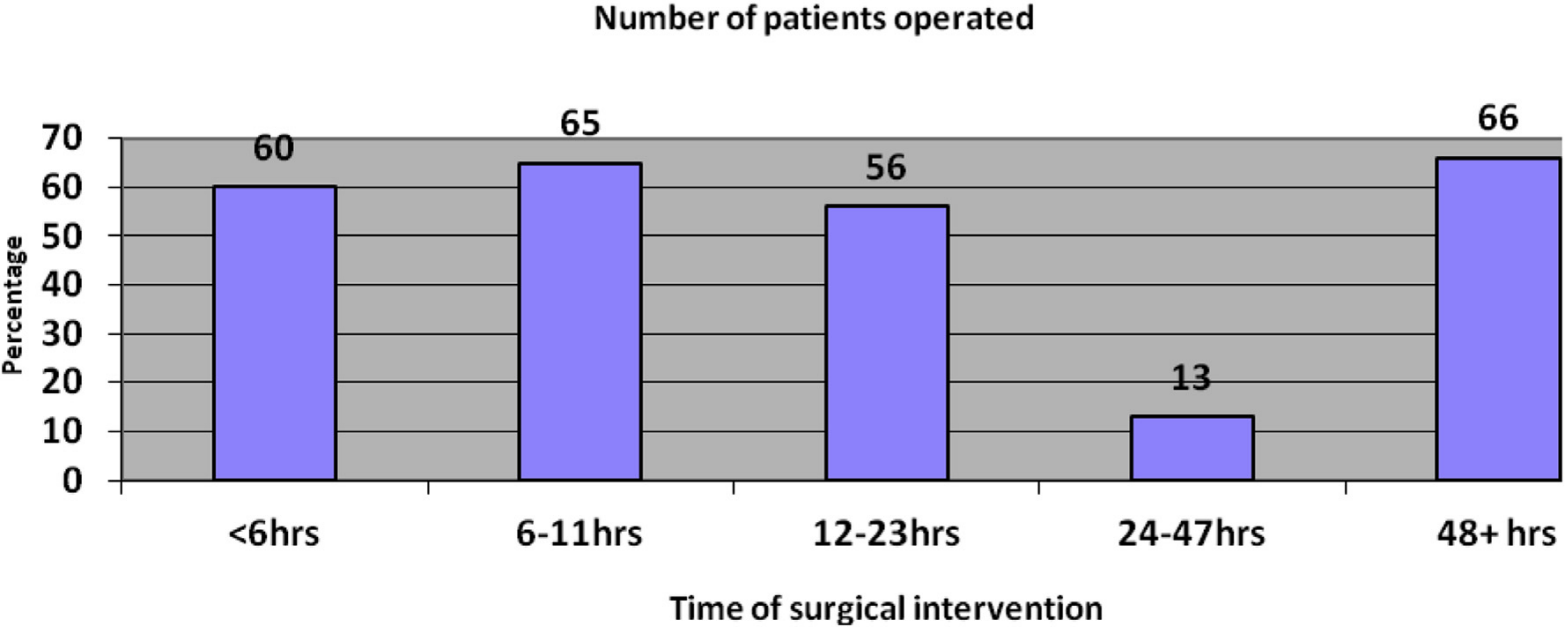
Time of surgical intervention after hospital admission, 2015.

Most patients had also late surgical intervention (>6hrs) after hospital admission according to the definition of the International Society of Emergency Laparotomy Network which is claimed to be attributing to the poor postoperative patient outcome [Fig. 2].

Most patients were given antibiotics prophylaxis before operation (n = 236, 90.8%). But only one out of 260 patients was given thromboembolic prophylaxis (chemical only) before operation. The majority of patients (n = 159, 61.2%) were operated during the night time. There was no the use of WHO or equivalent surgical safety checklist during the study period. The maximum, minimum and mead duration of operation were 360, 25 and 68.89 min respectively. The main surgical indications and type of operations performed are summarized below (Table 4, Table 5).

**Table 4:** Surgical indications (underlying pathology).

Indications (underlying pathology)	Frequency (n)	Percentage (%)
Penetrating trauma	32	12.3
Blunt trauma	14	5.4
Small bowel obstruction	18	6.9
Gangrenous small bowel	7	2.7
Ischemic small bowel	1	0.4
Large bowel obstruction	12	4.6
Malignancy	7	2.7
Peritonitis	15	5.8
Redundant sigmoid volvulus	4	1.5
Gangrenous sigmoid volvulus	8	3.1
Gastric perforation	6	2.3
Gangrenous right sigmoid colon	8	3.1
Perforated gastric ulcer	6	2.3
Intussusception	6	2.3
Abdominal abscess and adhesion	9	3.5
Acute appendicitis	87	33.5
Appendicial abscess	12	3.8
Negative laparotomy	8	3.1

**Table 5:** Types of operations performed.

Primary operations performed	Frequency (n)	Percentage (%)
Abdominal: Laparotomy plus		
Explorative laparotomy	38	14.6
Repair of perforated bowel	14	5.4
Bowel resection and anastomosis	36	13.8
Graham's patch	5	1.9
Hartmann's procedure	8	3.1
Colostomy	12	4.6
Abscess drainage and adheniolysis	19	7.3
Partial gastrectomy	12	4.6
Derotation	10	3.8
Splenectomy	4	1.5
Right hemicolectomy	3	1.2
Appendicial procedures		
Appendectomy	87	33.5
Abscess drainage & appendectomy	12	4.6

### 3.4 Postoperative patient management

**Fig. 3:**
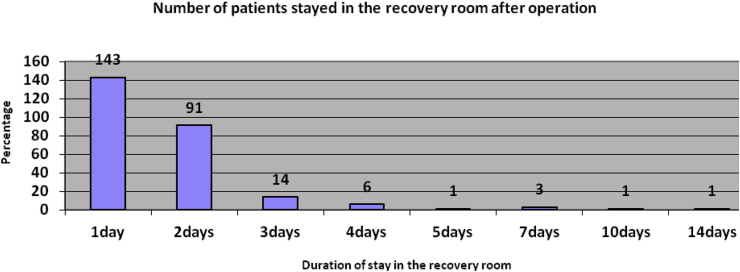
The duration of patient stay in the recovery room after operation, 2015.

Most patients passed through the recovery room after operation (n = 258, 99.2%). Only two patients directly transferred from the operation theatre to the ward and/or ICU [Fig. 3].

The majority of patients were managed in the surgical ward 103 (39.6%), trauma unit 98 (37.7%), orthopedics 38 (14.6%), paediatrics 19 (7.3%) and other 2 (0.8%) respectively. The anaesthetists involved in the postoperative patient management in 97 patients (37.3%). The minimum and maximum duration of the total length of hospital stay after operation was 1 and 30 days respectively with the median value of 6.0 ± 4.68 days.

### 3.5 Postoperative morbidity and associated factors

The overall incidence of postoperative morbidity was 39.2% (102/260) within 30 days of operation. Twenty six (10%) out of 260 patients were re-admitted from the wards to the recovery room after operation. Surgical re-intervention after operation was done for 14 (5.4%) patients. Of these, 11 (4.2%) under general anaesthetics, 2 (0.8%) under local anaesthetics and 1 (0.4%) endoscopic interventions were done.

The most common postoperative morbidity was vital sign derangement (n = 65, 25%) among patients who underwent emergency laparotomy with diagnosis of peritonitis (n = 11), penetrating trauma (n = 17), small bowel obstruction (n = 14), gastric perforation (n = 6), intussusception (n = 3), abdominal abscess (n = 5), perforated gastric ulcer (n = 3), gangrenous bowel (n = 2, 3.1%), ischemic bowel (n = 1, 1.5%) and large bowel obstruction (n = 2) respectively. In addition, pneumonia occurred in patients with penetrating trauma (n = 2), abdominal abscess (n = 3), gastric ulcer (n = 2), blunt trauma (n = 1) and negative laparotomy (1) respectively. Patients who developed wound infection were intussusception (n = 1), gangrenous sigmoid volvulus (n = 2), gangrenous right sigmoid colon (1) and blunt trauma (n = 1) respectively [Table 5].

**Table 6:** Factors related with incidence of postoperative morbidity (n = 102), 2015.

Factor	Frequency	Percentage (%)
Vital sign derangement	65	25
Hospital acquired pneumonia	10	3.8
Postoperative nausea and vomiting	6	2.3
Wound infection	5	1.9
Intra-abdominal abscess	5	1.9
Fever	5	1.9
Anastomotic leak	3	1.2
Abdominal distension	3	1.2
Intra-abdominal bleeding	1	0.4
Diarrhea	1	0.4

The variables with a p-value of <0.05 from the bivariate analysis but had no association with postoperative morbidity from the multivariate analysis were age, sex, history of diabetes mellitus, premedication, anaesthetists opinion about postoperative patient outcome, type of anaesthesia, level of consciousness during recovery from anaesthesia after operation, patient re-admission to the recovery room and perioperative blood transfusion. Consultant surgeons were involved in 84 operations only [Table 6].

### 3.6 Postoperative patient mortality and associated factors

The overall incidence of postoperative mortality was 3.5% (9/260). Of these, 3, 4 and 2 patients dead within 24 h, within 72 h and within 30 days after operation respectively. The variables with a p-value of <0.05 from the bivariate analysis but had no association with postoperative mortality from the multivariate analysis were age, sex, ASA status, co-morbidity, V/S at admission, preop analgesia, type of anaesthesia, intraoperative analgesia, type of muscle relaxant, V/S during recovery phase, time from admission to operation, type of operation, prophylactic antibiotics, use of intraoperative warming, and perioperative blood transfusion. Preoperative anaesthetists' opinion has positive association with postoperative mortality after laparatomy [Table 7] (see Table 8).

**Table 7:** Factors associated with postoperative morbidity (N = 260), 2015.

Variable	Frequency	AOR	95% CI	P-value
Surgeon				
Yes, consultant involved	84	0.404	0.201–0.812	0.011
Yes, senior resident with consultation	176	1		
Senior anaesthetist involved during operation				
Yes	43	0.417	0.179–0.970	0.042
No	217	1		
Preop co-morbidity				
No	227	0.383	0.156–0.939	0.036
Yes	35	1		
Anaesthetist involved in postoperative Mx				
Yes	97	3.364	1.801–6.282	0.000
No	163	1		
Bowel resection				
No	222	0.232	0.091–0.591	0.002
Yes	38	1

**Table 8:** Factors associated with postoperative mortality (N = 260), 2015.

Variable	Frequency	AOR	95% CI	P-value
Premedication				
No	141	12.068	1.052–137.624	0.045
Yes	19	1		
Consciousness level during recovery from anaesthesia				
Fully awake	213	0.114	0.021–0.628	0.013
Half awake	47	1		
Any 30 day re-intervention				
No	246	0.083	0.009–0.750	0.027
Yes	14	1		

## 4 Discussion

This study revealed that the overall incidence of postoperative morbidity and mortality were 39.2% (102/260) and 3.5% (9/260) within 30 days of operation respectively. This finding was high compared with a study conducted in Pakistan where the incidence of postoperative complication was 33.7%. This discrepancy could be due to better perioperative care of patients in Pakistan compared to our setup [11]. However, our finding was low compared with a study conducted in India [12] which could attribute to the difference in the quality of perioperative patient care.

The factors that had strong association with postoperative morbidity were presence of preoperative co-morbidity (*P* = 0.036), and bowel resection (*P* = 0.002) [13]. The presence of co-morbidities and extensive operations like bowel resection where patients mostly develop bowel ischemia/gangrene are well known factors contributing for postoperative complications after emergency laparotomy [9].

In addition, in the current study, the level of consciousness at the end of anaesthesia (P = 0.013) and any 30 day surgical re-intervention (*P* = 0.027) had positive association with postoperative mortality. Optimal perioperative patient care and early interventions could reduce postoperative patient mortality [14].

Concerning postoperative morbidity, the commonest postoperative complications were vital sign derangement (n = 65, 25%), hospital acquired pneumonia (n = 10, 3.8%), PONV (n = 6, 2.3%), wound infection (n = 5, 1.9%), intra-abdominal abscess (n = 5, 1.9%), fever (n = 5, 1.9%) and anastomotic leak (n = 3, 1.2%) respectively. The incidences of pneumonia and wound infection were low in our study compared with a previous study which might attribute to the quality of perioperative surgical and anaesthetic care provision [14].

Moreover, late presentation of the patients to the hospital and delay surgical intervention after admission to the hospital contributes greatly for perioperative patient morbidity and mortality [2,15]. In the current study, the majority of patients (n = 249, 95.7%) had late (>6hrs) presentation to the hospital after the onset of symptoms of the diseases and late surgical intervention (>6hrs) after hospital admission (n = 200, 76.9%) respectively according to the definition of the International Society of Emergency Laparatomy Network [2,15]. This finding was comparable with a previous study [12]. The late presentation might be due to the fact that most of our patients came from rural areas and there were also large emergency case-loads to the hospital which could attribute to late surgical interventions.

Most patients passed through the recovery room after operation (n = 258, 99.2%). Only two patients directly transferred from the operation theatre to the ward and/or ICU. Moreover, there was no surgical ICU which could contribute for postoperative adverse outcomes as failure to admit patients to the appropriate level of care immediately after emergency laparatomy is the main cause for morbidity and mortality [16]. Furthermore, there was no the use of WHO or equivalent surgical safety checklist during the study period. The establishment of high dependency unit and the use of WHO or equivalent surgical safety checklist during operation may improve postoperative patient outcome after such high risk operations [[17], [18], [19], [20], [21]]. It is also agreed that high risk operations, emergency laparotomy, should be specialist surgeons and anaesthetists lead [22]. However, in this study, consultant surgeons and anaesthetist were involved only in the few numbers of patients during operation [23].

### 4.1 Limitation and strength of the study

This is an observational study where practice variations among caregivers (medical interns, nurses, residents, surgeons, anaesthetists) during the perioperative course of the patient care could affect the study outcomes. In addition, lack of use of WHO or equivalent surgical safety checklist and surgical ICU could negatively impact on the postoperative patient morbidity and mortality after emergency abdominal surgery. This is the first study on postoperative patient outcome after emergency laparotomy in the hosting hospital and country which could provide an insight about the significance of the existed problem and the need for developing perioperative patient care bundle.

## 5 Conclusion

The incidence of postoperative morbidity and mortality were high in our University tertiary teaching and referral hospital. Preoperative co-morbidity and bowel resection were determinant factors for postoperative morbidity whereas the level of consciousness during recovery from anaesthesia, and any re-intervention within 30 days after primary laparotomy operation were contributing factors for postoperative patient mortality. Preoperative optimization, early surgical intervention, and consultant-surgeon/anaesthetist lead perioperative care for these high risk surgical patients could improve postoperative outcome. In addition, WHO or equivalent centre based surgical safety checklist during operation and establishment of high dependency unit should be emphasized. Moreover, investigation modalities like CT scan need to be established in the hospital to improve the quality of preoperative diagnosis and perioperative surgical patient care. Furthermore, perioperative patient care bundle/protocol should be introduced in the hospital to improve patient safety. It will be also paramount conducting the same study in large cohorts of patients in similar settings in the country.

## Provenance and peer review

Not commissioned, externally peer reviewed.

## Ethical approval

Ethical approval was obtained from University of Gondar, College of Medicine and Health Sciences, Academic, Research and Community Services Vice Dean (Ref.No.CMHS248/07). Please see the attached ethical clearance file.

## Sources of funding

This study was supported by University of Gondar (money used for questionnaire duplication and paid for data collectors). This grant had no influence on the conduct of study and manuscript preparation.

## Author contribution

Endale Gebreegziabher Gebremedhn, Abatneh Feleke Agegnehu and Bernard Bradley Anderson conceived the study, developed the proposal, collected data, analyzed data, prepared the manuscript, approved the final manuscript and agreed to publish in International Journal of Surgery.

## Conflicts of interest

No conflicts of interest to declare.

## Research Registration Number

Research Registration Unique Identifying Number (UIN): researchregistry3317

## Guarantor

None.

## Consent

N/A

## Appendix A Supplementary data

Process Checklist-revised Process Checklist-revised
